# Emergence of Extensively Drug-Resistant and Hypervirulent KL2-ST65 Klebsiella pneumoniae Harboring *bla*_KPC-3_ in Beijing, China

**DOI:** 10.1128/spectrum.03044-22

**Published:** 2022-11-14

**Authors:** Chao Liu, Pengcheng Du, Ping Yang, Juan Yi, Ming Lu, Ning Shen

**Affiliations:** a Department of Infectious Disease, Peking University Third Hospital, Beijing, China; b Center of Infectious Disease, Peking University Third Hospital, Beijing, China; c Qitan Technology Ltd., Chengdu, China; d Department of Pulmonary and Critical Care Medicine, Peking University Third Hospital, Beijing, China; e Institute of Medical Technology, Peking University Health Science Center, Beijing, China; Universidad de Buenos Aires, Facultad de Farmacia y Bioquímica

**Keywords:** multidrug-resistant hypervirulent *Klebsiella pneumoniae*, ST65, *bla*_KPC-3_

## Abstract

Multidrug-resistant hypervirulent Klebsiella pneumoniae (MDR-hvKp) has been emerging worldwide. However, the clinical, microbiological, and genomic characteristics of newly emerged MDR sequence type 65 (ST65) hvKp are unclear. We conducted active longitudinal genomic surveillance of *K. pneumoniae* in the hospital starting in 2017. Clinical characteristics, including demographic data, infection type, and outcomes, were collected. Whole-genome sequencing was performed to clarify phylogenetic and plasmid features, and phenotype determined by growth curves, plasmid transferability and stability, hypermucoviscosity, biofilm formation, and serum survival were analyzed to microbiologically characterize ST65 in depth. Ten ST65 (1.4%, 10/720) isolates were detected from 720 *K. pneumoniae* isolates in total. Nine patients (90%, 9/10) were older than 60 years and had multiple underlying diseases. All ST65 *K. pneumoniae* isolates harbored *iucA*, *rmpA*, *rmpA2*, *iroB*, and *peg344* and were identified as hvKp. Surprisingly, two MDR-hvKp isolates that grew slowly were observed. Isolate PEKP4222 harbored a pLVPK-like plasmid and a conjunctive MDR plasmid. Isolate P1 harbored *bla*_KPC-3_ in a new plasmid, pP1-54, resulting in an extensively drug-resistant (XDR) phenotype; this isolate, which might have evolved from a strain harboring *bla*_KPC-2_, resulted in fatal infection. The pP1-54 plasmid could not be transferred to Escherichia coli by conjugation but could be stably inherited vertically. Interestingly, P1 also carried the pLVPK-like plasmid and acquired various antimicrobial resistance genes, and *bla*_CTX-M-3_ was detected in the IncB/O/K/Z plasmid. The convergence of XDR and hypervirulence within classical ST65 hvKp is emerging, highlighting the need for enhanced genomic surveillance.

**IMPORTANCE** XDR-hvKp poses a great challenge to public health. ST65, a classical hvKp subtype, mostly presented with hypermucoviscosity, which restricts antimicrobial resistance acquisition. However, few studies have demonstrated the clinical, microbiological, and genomic characteristics of ST65, especially MDR-ST65 hvKp. Here, we first reported that ST65 hvKp acquired *bla*_KPC-3_ and then conferred the XDR-hvKp phenotype. Genomic context analysis concluded that the *bla*_KPC-3_ gene might have evolved from *bla*_KPC-2_. Additionally, the pLVPK-like plasmid seemed to acquire more resistance genes, and *bla*_CTX-M-3_ located in the IncB/O/K/Z plasmid was observed. The XDR-hvKp phenotype could be stably inherited vertically, indicating that strains harboring *bla*_KPC-3_ and pLVPK-like plasmids could persistently exist in hospital settings. These data suggest that genomic adaptation is rapid and that enhanced surveillance is essential.

## INTRODUCTION

Klebsiella pneumoniae is emerging as an important pathogen in clinical practice due to the acquisition of external genes (1). Since hypervirulent Klebsiella pneumoniae (hvKp) was first reported in Taiwan, *K. pneumoniae* has gradually evolved into two pathotypes: hvKp and classical *K. pneumoniae* (cKp) (2). However, an increasing number of epidemiological investigations of hvKp suggest that hvKp might be replacing cKp as the dominant pathotype in high-risk settings (3).

Multilocus sequence type 65 (ST65), a typical hvKp subtype, is generally sensitive to most antimicrobial agents and can cause fatal infections (4). Its isolation followed that of the most common sequence type, ST23 hvKp, which results in fatal invasive infection in different countries and regions. Unfortunately, the rate of convergence of multidrug resistance and hypervirulence within *K. pneumoniae* (MDR-hvKp) is rapidly increasing, posing a great challenge to public health (2, 5, 6). Several common hvKp subtypes, such as ST23 and ST86, present higher resistance levels and have evolved into MDR-hvKp. Our previous study elucidated that the evolution of MDR-hvKp was primarily due to the acquisition of key virulence genes within cKp (3). Most hvKp strains possess a thick capsule that serves as a barrier for the uptake of external genes (7). However, the typical hvKp strain, ST23, tends to acquire antimicrobial resistance genes via the plasmid and IS*26*, resulting in MDR-hvKp (8). Interestingly, *bla*_CTX-M-like_, not *bla*_KPC_ or *bla*_NDM_, is common in MDR-ST23-hvKp (8), which is different from the ST11 endemic clone (9). To date, research on MDR-hvKp within ST65 has been extremely scarce. A previous study reported *bla*_CTX-M-like_ (10), *bla*_IMP-4_ (11), and *bla*_NDM_ (12) in ST65 hvKp strains. However, few studies have focused on the systematic clinical, microbiological, and genomic characteristics of ST65 hvKp strains in China.

*bla*_KPC-2_ is common among *K. pneumoniae* strains (13). However, the endemic vector is different between China (ST11) (13) and the Occident (ST258) (14). *bla*_KPC-3_ has a single amino acid substitution (H272Y) and is distinguishable from *bla*_KPC-2_, which confers efficient hydrolysis of cephalosporins and carbapenems (15, 16). Interestingly, both *bla*_KPC-3_ and *bla*_KPC-2_ seem to be prevalent in the United States and Europe, whereas *bla*_KPC-3_ is rare in China (16).

Here, we report for the first time the emergence of extensively drug-resistant (XDR) ST65 hvKp harboring *bla*_KPC-3_ and *bla*_CTX-M-3_ in a hospital during approximately 5 years of genomic surveillance. Furthermore, the pLVPK-like plasmid acquired *arr-3* and *bla*_OXA-1_ encoded by a single ~24-kb resistance region flanked by IS*26*. These findings are concerning and have significant implications for enhanced genomic surveillance.

## RESULTS AND DISCUSSION

### Clinical and microbiological characterization.

**FIG 1:**
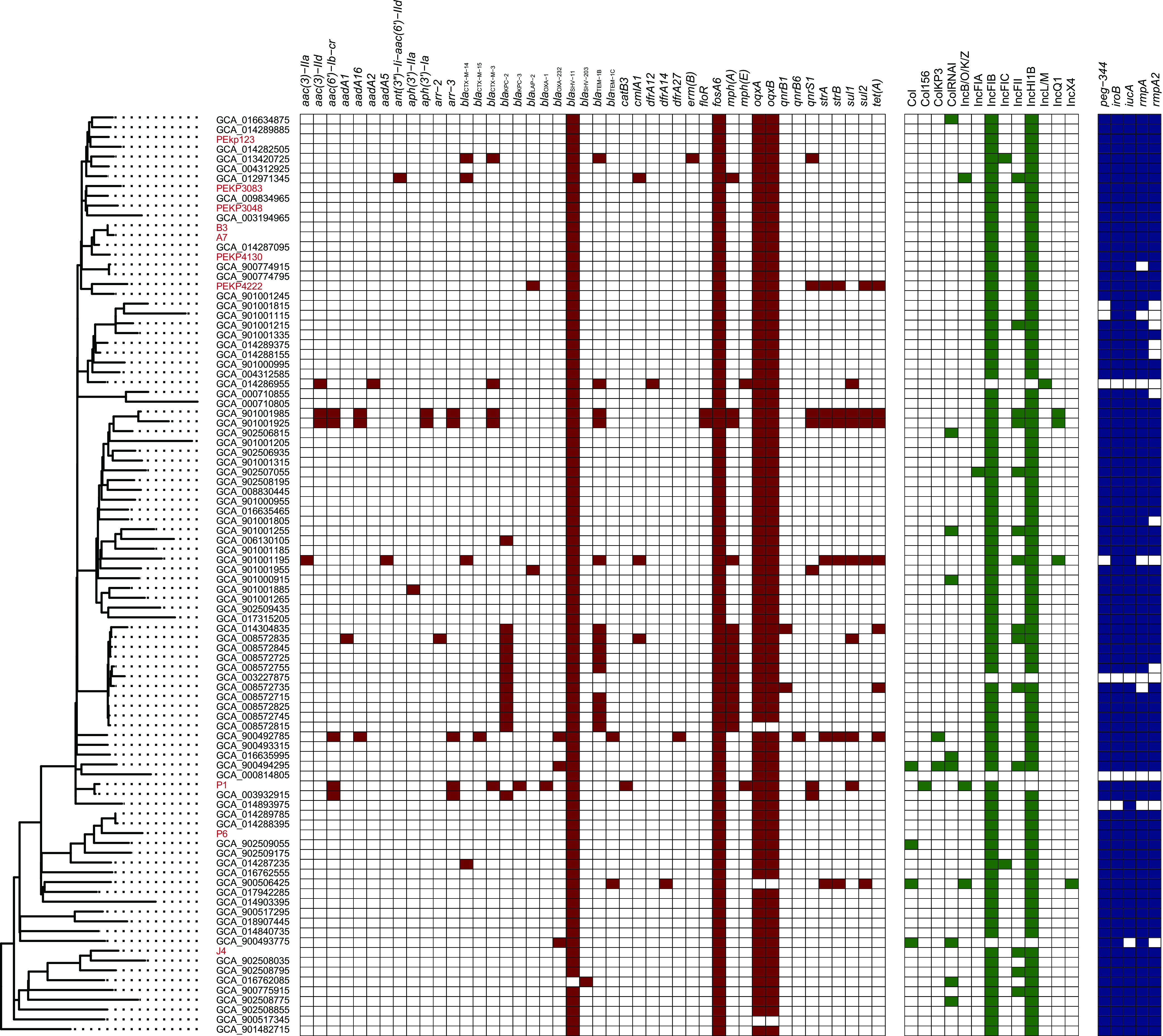
Global microbiological characteristics of the ST65 strains. Isolates isolated from our surveillance are in red.

During the 4-year study period of continuous surveillance in the hospital, 10 ST65 *K. pneumoniae* (1.4%, 10/720) isolates were identified. All the ST65 isolates presented a hypermucoviscous phenotype, possessed a combination of five key virulence genes, and were identified as hvKp. All the ST65 isolates presented with the KL2 serotype and O1v2, whereas four of them represented serum resistance (Table 1, Fig. 1). Interestingly, half of the infections caused by ST65 (50%, 5/10) were defined as hospital-associated infections (HAIs) (Table 2), suggesting that ST65 hvKp might also become one of the hvKp subtypes responsible for nosocomial infection, as previously described (3). In addition, we also calculated the nucleotide differences among the isolates. There were only 11 core genome single nucleotide polymorphism (SNPs) (cgSNPs) between isolate A7 and B3, indicating potential transmission between the two patients infected by the two isolates (see Fig. S3 in the supplemental material). However, although 8 isolates were phylogenetically clustered together, except J4 and P6, 60 to 151 cgSNPs were identified among these isolates, except A7 and B3, showing indirect transmission among these patients. The ST65 isolates might instead remain in the hospital environment or be carried by asymptomatic patients and cause infections occasionally.

**TABLE 1:** Microbiological and genomic characteristics of the ST65 strains

Item^*a*^	P1	PEKP4222	P6	PEKP4130	B3	PEKP3083	PEKP3048	PEKP123	A7	J4
HMV	**Y**	**Y**	Y	Y	Y	Y	Y	N	Y	Y
MDR	**Y**	**Y**	N	N	N	N	N	N	N	N
CR	**Y**	**N**	N	N	N	N	N	N	N	N
Biofilm	+	+	+	+	+	+	+	++	+	+
Serum killing assay	Sensitive	Sensitive	Sensitive	Resistant	Resistant	Sensitive	Resistant	Sensitive	Sensitive	Resistant
AMR	***bla*_OXA-1_; *bla*_CTX-M-3_; *bla*_KPC-3_**	***tetA***	N	N	N	N	N	N	N	N
Serotype	**KL2**	**KL2**	KL2	KL2	KL2	KL2	KL2	KL2	KL2	KL2
O-antigen	**O1v2**	**O1v2**	O1v2	O1v2	O1v2	O1v2	O1v2	O1v2	O1v2	O1v2
yersiniabactin	***Ybt17*-ICEKP10**	***Ybt17*-ICEKP10**	N	*Ybt17*-ICEKP10	*Ybt17*-ICEKP10	N	*Ybt17*-ICEKP10	*Ybt17*-ICEKP10	*Ybt17*-ICEKP10	N
Colibactin	***clb3***	***clb3***	N	*clb3*	*clb3*	N	*clb3*	*clb3*	*clb3*	N
Aerobactin	***iuc1***	***iuc1***	*iuc1*	*iuc1*	*iuc1*	*iuc1*	*iuc1*	*iuc1*	*iuc1*	N
Salmochelin	***iro1***	***iro1***	*iro1*	*iro1*	*iro1*	*iro1*	*iro1*	*iro1*	*iro1*	*iro1*
RmST	**38**	**38**	38	38	38	38-1LV	38	38	38	38
Virulence score^*b*^	**5**	**5**	3	5	5	3	5	5	5	3

a HMV, hypermucoviscosity; MDR, multidrug resistance; CR, carbapenem resistance; AMR, antimicrobial resistance; Y, yes; N, no.

b Virulence score was predicted using Kleborate. Bold texts represent the characteristics of the two MDR-ST65-hvKp strains.

**TABLE 2:** Clinical characteristics of the ST65 *K. pneumoniae* infected patients^*a*^

Patient	A7 and B3	J4	P1	P6	PEKP3048	PEKP3083	PEKP4130	PEKP4222	PEKP123
Age (yrs)	62	64	**82**	91	73	86	63	**90**	55
Sex	Male	Male	**Male**	Male	Male	Male	Male	**Male**	Female
Specimen	Pus	Sputum	**Urine**	Sputum	Sputum	Sputum	Sputum	**Sputum**	Blood
Collection date (yr/mo/day)	2021/1/13	2021/2/18	**2017/11/15**	2017/1/6	2020/11/23	2020/12/26	2021/3/10	**2017/1/3**	2020/11/23
Department	Wound treatment center	Neurology	**ICU**	Geriatrics	Cardiac surgery	Emergency	Respiratory medicine	**Geriatrics**	Emergency
Underlying diseases	Diabetes mellitus, neck abscess	Diabetes mellitus, stomach ulcer, hepatitis B	**Parkinson’s, cerebral infarction, hypertension, hepatitis, duodenal ulcer with bleeding, prostatic hyperplasia**	Gastric lipoma, atrial fibrillation, colon cancer	Hyperlipidemia, cerebral infarction, reflux esophagitis, trauma to the right eye, hernia repair	Cerebral infarction, atrial fibrillation, pneumonia, diabetes	Coronary heart disease, left carotid artery stenosis, peripheral atherosclerosis, hyperlipidemia, fatty liver, reflux esophagitis, chronic inflammation of the gastric mucosa with erosions, low-grade tubular adenoma of the colonic mucosa	**Diabetes mellitus, reflux esophagitis, chronic renal insufficiency, multiple atherosclerotic plaques of the carotid arteries, old pulmonary tuberculosis, lumbar disc herniation**	Hypertension, systemic lupus erythematosus, interstitial lung disease, chronic superficial gastritis with erosions, duodenoccalitis, multiple osteonecrosis of the bilateral femur and tibia
Antibiotic agent exposure	N	N	**Minocycline; imipenem**	N	N	N	N	**Moxifloxacin; ceftazidime**	N
Incubation	N	Gastrostomy tube	**Gastrostomy tube, urinary catheter, CVC, tracheostomy tube**	N	N	Gastrostomy tube, urinary catheter	N	**N**	N
Infection type	CAI	HAI	**HAI**	HAI	HAI	CAI	CAI	**HAI**	CAI
Metastatic infection	N	N	**Y**	N	N	N	N	**N**	N
Mechanical ventilation	N	N	**Y**	N	N	N	N	**N**	N
Vasoactive drugs	N	N	**Y**	N	N	N	N	**N**	N
CCI	1	3	**5**	3	3	3	4	**5**	4
SOFA		0	**7**	0	3	3	2	**5**	
Outcome in 30 days	Survive	Survive	**Death**	Survive	Survive	Survive	Survive	**Survive**	Survive

a CVC, central venous catheter; HAI, hospital-acquired infection; CAI, community-acquired infection; CCI, Charlson comorbidity index; SOFA, sequential organ failure assessment; Y, yes; N, no. Bold texts represent the characteristics of the two MDR-ST65-hvKp strains.

Notably, 90% (9/10) of the patients infected with ST65 isolates were male. Additionally, most of the patients (90%, 9/10) were older than 60 years and had multiple underlying diseases (Charlson comorbidity index ([CCI], ≥3) (Table 2). It seems that hvKp can trigger infection in the elderly population (17, 18), challenging previous reports stating that hvKp is highly associated with infection in immunocompetent young adults (5, 19). Most of the isolates (80%, 8/10) were from sputum, and 20% of the isolates were from sterile body fluids (Table 2), similar to findings in previous studies that showed that hvKp was common in the respiratory system (3, 17, 18, 20, 21).

Importantly, two MDR-ST65 hvKp isolates (P1 and PEPK4222) emerged in the hospital. Strain P1 harbored *bla*_KPC-3_ and *bla*_CTX-M-3_, which conferred the XDR phenotype, resulting in treatment failure and fatal infection. Additionally, the plasmid possessing *bla*_KPC-3_ could not be successfully transferred into Escherichia coli but did exhibit stable inheritance (Table 3). The other isolate, PEKP4222, harbored various genes conferring quinolone resistance, suggesting that active genomic surveillance is essential. The MDR-ST65 hvKp isolates (P1 and PEPK4222) did not show significant differences in their growth curves but proliferated more slowly than the other ST65 hvKp isolates (Fig. S1). The fitness cost of MDR-ST65 hvKp is notable.

**TABLE 3:** Antibiotic resistance patterns of the ST65 clone and its passage^*a*^

Antibiotic	AST data for (mg/L):											
	P1	PEKP4222	P6	PEKP4130	B3	PEKP3083	PEKP3048	PEKP123	A7	J4	10th passage–P1	10th passage–PEKP4222
Ticarcillin/clavulanate	≥128	≤8	≤8	≤8	≤8	≤8	≤8	≤8	≤8	≤8	≥128	≤8
Piperacillin/tazobactam	≥128	8	≤4	≤4	≤4	≤4	≤4	≤4	≤4	≤4	≥128	8
Ceftazidime	≥64	0.5	≤0.12	0.25	≤0.12	≤0.12	≤0.12	0.25	≤0.12	≤0.12	≥64	0.5
Cefoperazone/sulbactam	≥64	≤8	≤8	≤8	≤8	≤8	≤8	≤8	≤8	≤8	≥64	≤8
Cefepime	≥32	≤0.12	≤0.12	≤0.12	≤0.12	≤0.12	≤0.12	≤0.12	≤0.12	≤0.12	≥32	≤0.12
Aztreonam	≥64	≤1	≤1	≤1	≤1	≤1	≤1	≤1	≤1	≤1	≥64	≤1
Imipenem	≥16	≤0.25	≤0.25	≤0.25	≤0.25	0.5	0.5	≤0.25	0.5	≤0.25	≥16	0.5
Meropenem	≥16	≤0.25	≤0.25	≤0.25	≤0.25	≤0.25	≤0.25	≤0.25	≤0.25	≤0.25	≥16	≤0.25
Amikacin	≥64	≤2	≤2	≤2	≤2	≤2	≤2	≤2	≤2	≤2	≥64	≤2
Tobramycin	≥16	≤1	≤1	≤1	≤1	≤1	≤1	≤1	≤1	≤1	≥16	≤1
Ciprofloxacin	≥4	2	≤0.25	≤0.25	≤0.25	≤0.25	≤0.25	≤0.25	≤0.25	≤0.25	≥4	2
Levofloxacin	≥8	4	≤0.12	≤0.12	≤0.12	≤0.12	≤0.12	≤0.12	≤0.12	≤0.12	≥8	4
Doxycycline	≥16	≥16	1	1	1	1	1	1	1	1	≥16	≥16
Minocycline	8	≥16	≤1	≤1	≤1	≤1	≤1	2	≤1	≤1	≥16	≥16
Tigecycline	2	≥8	≤0.5	≤0.5	≤0.5	≤0.5	≤0.5	1	≤0.5	≤0.5	2	≥8
Trimethoprim/sulfamethoxazole	40	≥320	≤20	≤20	≤20	≤20	≤20	≤20	≤20	≤20	40	≥320
Ceftazidime/avibactam*b*	22	27	27	29	28	28	26	25	26	26	21	21
Cefiderocol*b*	22	28	27	27	27	27	28	26	24	26	21	27

a AST was conducted using the K-B method.

### Genomic characteristics of ST65 *K. pneumoniae*.

We also collected 84 ST65 *K. pneumoniae* genomes to further investigate the genomic characteristics, including resistance genes and virulence gene profiles, of ST65. Almost all of the ST65 isolates represented KL2, which was initially identified as a biomarker of hvKp. In addition, most of the isolates (96.8%, 91/94) were confirmed as O1v2 for the O locus, while two were O2v2, and one was O1v1. Among the different O loci within the *K. pneumoniae* strains, O2v2 presented with a lower 30-day mortality rate (22). The other O loci, such as O1v2, that emerged in the study, should be sources of concern. The presence of *iroB*, *iucA*, *peg344*, *rmpA*, and *rmpA2* together is highly accurate for the identification hvKp, and we found that most of the ST65 genomes (83.0%, 78/94) harbored these five key virulence genes, suggesting that ST65 is a classic hvKp possessing a pLVPK-like plasmid, similar to ST23 (8). Among our 10 isolates, although the 5 above-described virulence genes were all positive, 3 isolates, P6, J4, and PEP3083, were negative for *clb* and *ybt* genes and received a virulence score of 3 with Kleborate software, whereas the other 7 isolates with *clb* and *ybt* genes had a score of 5. We analyzed the differences of string tests, sensitivity to serum killing, and the ability to form biofilm; however, no significant differences were observed, which might due to the small number of isolates (Tables S1, S2, S3, S4, and S5).

Moreover, most of the ST65 genomes harbored few resistance genes, except that *bla*_SHV-11_ (conferring resistance to beta-lactams), *fosA6* (conferring resistance to fosfomycin), and *oqxAB* (conferring resistance to quinolones) were harbored by most of the genomes and positive in all of our isolates. However, *fosA* and *oqxAB* do not necessarily confer fosfomycin and quinolone resistance in K. pneumoniae. Among our isolates, the resistance rate of quinolones was 20% (2/10). These results indicate that detection of these resistances cannot rely on molecular tests of the corresponding genes, but should rely on the phenotypic tests. Among the 94 strains, 19 (20.2%, 19/94) were predicted to be MDR, harboring resistance genes conferring resistance to three or more different antimicrobial classes. The majority (13/19, 68.4%) of MDR strains were associated with *bla*_KPC_, including 12 strains harboring *bla*_KPC-2_ from published data and 1 isolate harboring *bla*_KPC-3_ obtained in this study (Fig. 1). Among our 10 isolates, isolates P1 and PEKP4222 were predicted as XDR and MDR, respectively. P1 harbored resistance genes including *bla*_OXA-1_, *bla*_CTX-M-3_, *bla*_KPC-3_, *aac(6′)-Ib-cr*, and *qnrS1* and correspondently showed resistance to penicillins, cephalosporins, carbapenems, aminoglycosides, quinolones, and tetracyclines. PEKP4222 harbored resistance genes including *qnrS1*, *tet*(A), and *sul2* and showed resistance to quinolones, tetracyclines, and trimethoprim/sulfamethoxazole.

### Characteristics of a novel plasmid harboring *bla*_KPC-3_.

**FIG 2:**
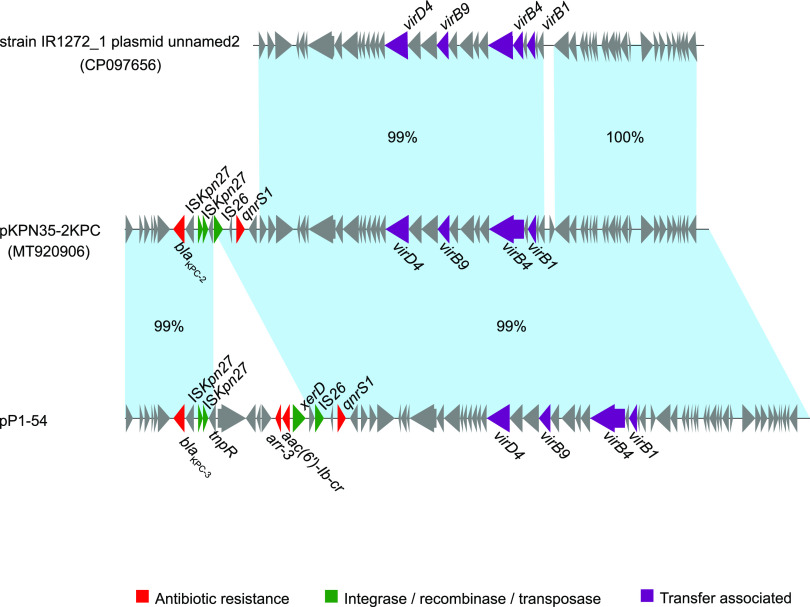
Linear plasmid sequence comparison of plasmids pP1-54 and pKPN35-2KPC and strain IR1272_1 plasmid unnamed2. The matched regions between two sequences are displayed by light blue blocks. The arrows represent the genes related to resistance and transfer (red, resistance genes; green, integrase, recombinase, and transposase genes; purple, transfer-associated genes; gray, genes of other functions).

We obtained the complete sequence of *bla*_KPC-3_-positive ST65 isolate P1. The *bla*_KPC-3_ gene was harbored by a 54-kb plasmid named pP1-54. pP1-54 also represented *qnrs1*, *arr-3*, and *aac(6′)-Ib-cr* and comprised unknown plasmid replicons. Plasmid pP1-54 is similar to plasmid pKPN35-2KPC (accession number MT920906, 85.3% coverage with 99.0% identity), which is 46 kb in size and comes from a *K. pneumoniae* strain also isolated in Beijing, China (Fig. 2). Compared with plasmid pKPN35-2KPC, an ~8-kb fragment harboring *arr-3* and *aac(6′)-Ib-cr* was inserted into IS*26*. Interestingly, plasmid pKPN35-2KPC harbored *bla*_KPC-2_ instead of *bla*_KPC-3_. Additionally, the plasmid pP1-54 is likely an IncX6 plasmid named MT920906, isolated from Enterobacter cloacae, suggesting the possibility that *bla*_KPC-3_ harboring pP1-54 was horizontally acquired (23).

A previous study reported that *bla*_KPC-2_ could insert into ST65 and ST23 hvKp in endemic *bla*_KPC-2_ settings (8, 24). Recently, Chen et al. (16) reported an outbreak of IncX8 type plasmid-mediated KPC-3-producing *Enterobacterales* infection in China, during which two ST65 hvKp strains were identified. In this study, we found a new plasmid harboring *bla*_KPC-3_ within ST65 hvKp that enriched the genomic context of *bla*_KPC-3_, suggesting that enhanced genomic surveillance is essential.

### Acquisition of resistance genes on the pLVPK-like plasmid.

**FIG 3:**
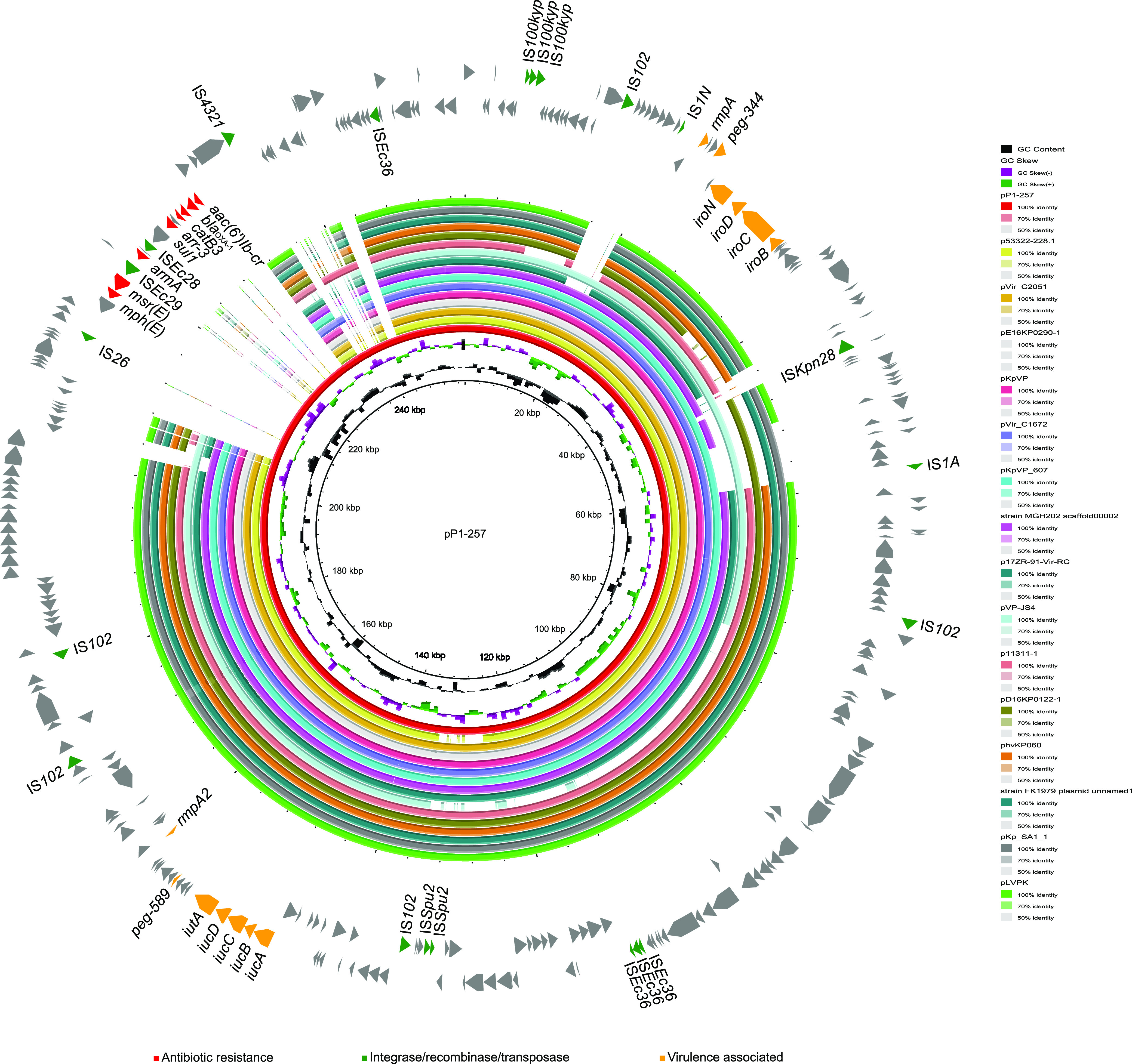
Comparison between plasmid pP1-257 found in this study and similar plasmids found in the online NCBI database. The outermost circle of arrows indicates the genes of reference plasmid pP1-257 used for comparison (red, resistance genes; green, integrase, recombinase, and transposase genes; orange, virulence-associated genes; gray, genes of other functions).

In addition to the *bla*_KPC-3_-possessing plasmid, an ~257-kb pLVPK-like plasmid was also identified in the P1 isolate, named pP1-257. Virulence-associated genes were identified on this plasmid, including *rmpA*, *rmpA2*, *pep-344*, *pep-589*, *iutA*, the *iuc* cluster, and the *iro* cluster, similar to the classic virulence plasmid pLVPK. This plasmid comprised the IncFIB/IncHI1B type of plasmid replicon and resistance genes, including *bla*_OXA-1_, *armA*, *aac(6′)Ib-cr*, *catB3*, *arr-3*, *sul1*, *msr*(E), and *mph*(E) (Fig. 3). Notably, these resistance genes were encoded by a single ~24-kb resistance region, with IS*26* on one side, which was considered an insertion fragment compared with plasmid pLVPK. This insertion accounted for the MDR phenotype, including β-lactamase, aminoglycoside, macrolide, etc., resistance (Table 3). These results indicated that this insertion converted the virulence plasmid to an MDR virulence plasmid, which might be a more serious threat to patients.

A previous study reported two main mechanisms of MDR-hvKp development (2, 6). One is the acquisition of key virulence-associated genes in cKp (3, 25). The other is the acquisition of various resistance genes/plasmids in hvKp (8). In this study, we found that ST65 hvKp had obtained both resistance genes and MDR plasmids that conferred the MDR-hvKp phenotype, highlighting the urgent need to enhance surveillance to prevent dissemination. In addition, IS*26* mediated the acquisition of resistance genes on both the MDR plasmid and virulence plasmid, indicating the wide impact of this element on resistance gene transfer.

### The genetic context of *bla*_CTX-M-3_ within ST65 hvKp strains.

**FIG 4:**
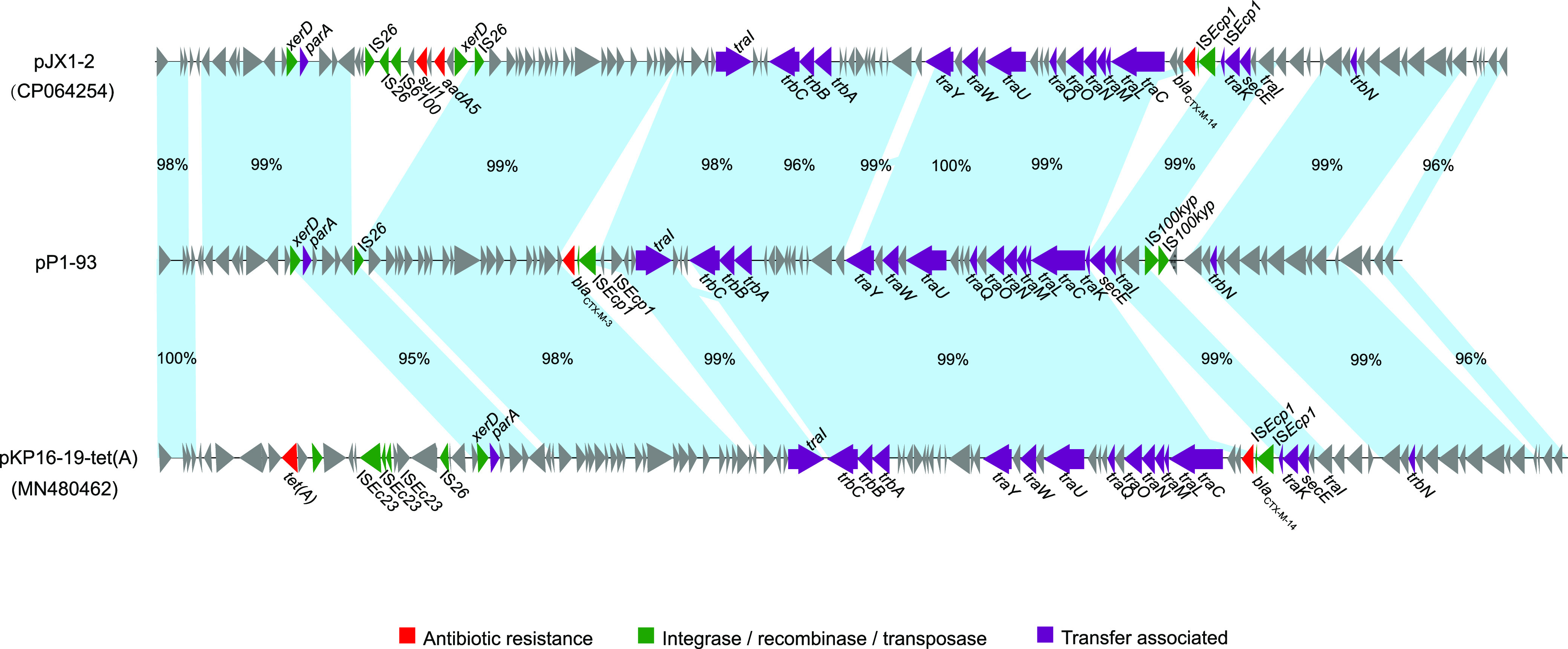
Linear plasmid sequence comparison of plasmids pP1-93, pJX1-2, and pKP16-19-tet(A). The matched regions between two sequences are displayed by light blue blocks. The arrows represent the genes related to resistance and transfer (red, resistance genes; green, integrase, recombinase, and transposase genes; purple, transfer-associated genes; gray, genes of other functions).

A ~93-kb plasmid named pP1-93, comprising IncB/O/K/Z possessing *bla*_CTX-M-3_, was also detected in the study. IncB/O/K/Z plasmid replication is common in *Enterobacteriaceae* isolated from animals and has shown an obvious increase in the 2020s (26). Plasmid pP1-93 is similar to plasmid pJX1-2 (accession number CP064254, 90.2% coverage and 98.6% identity) and plasmid pKP16-19-tet(A) (accession: MN480462, 81.1% coverage and 97.9% identity) from *K. pneumoniae* isolated from two patients in different provinces of China, indicating that this group of plasmids might be common in *K. pneumoniae* in China. The transposon comprising *bla*_CTX-M-3_ and IS*Ecp1* was detected on plasmid pP1-93, and those comprising *bla*_CTX-M-14_ and IS*Ecp1* were detected on the other two plasmids (Fig. 4).

**FIG 5:**
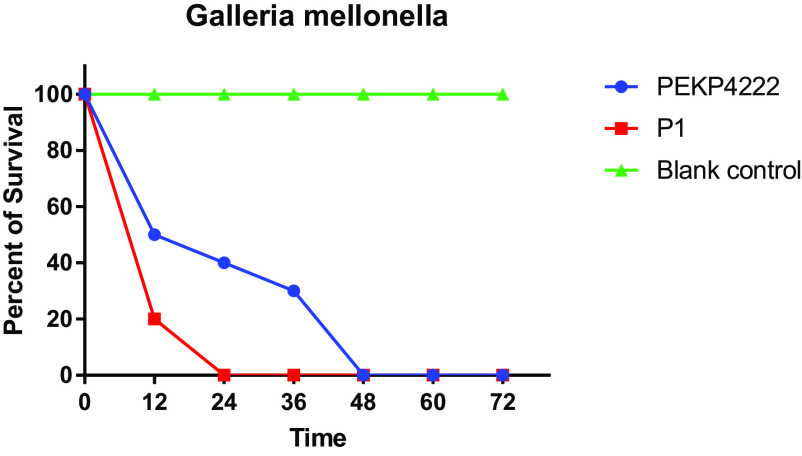
Virulence of the MDR-ST65-hvKp evaluated by the Galleria mellonella lethality assay.

A previous study reported that the emergence of ST65 resistance to cephalosporins was partly attributable to the coexistence of *bla*_CTX-M-3_ and *bla*_CTX-M-14_; cephalosporin-resistant ST65 was further identified as a non-biofilm producer with serum sensitivity and low virulence in the Galleria mellonella model (10). In this study, MDR-ST65 hvKp, P1, and PEKP4222 produced biofilms and presented hypervirulence *in vivo* but presented the inverse phenotype in the serum killing assay (Table 1, Fig. 5, Fig. S2), suggesting broad heterogeneity within the ST65 strains.

In conclusion, we report for the first time the acquisition of *bla*_KPC-3_ by classical hvKp ST65, conferring hypervirulence and the XDR phenotype. Interestingly, various resistance genes have been transferred to the pLVPK-like plasmid, and the genetic context of *bla*_CTX-M-3_ is diverse. XDR-ST65 hvKp, harboring *bla*_KPC-3_, *bla*_CTX-M-3_, and virulence-associated plasmids, can successfully propagate vertically, and enhancing genomic surveillance to prevent its rapid dissemination and evolution is essential.

## MATERIALS AND METHODS

### Included strains and their associated clinical characteristics.

Peking University Third Hospital has continuously conducted active genomic surveillance of *K. pneumoniae* since 2017. A total of 720 Klebsiella sp. strains are included in the database. All the strains were stored at −80°C. All the strains were first identified by matrix-assisted laser desorption ionization–time of flight (MALDI-TOF) mass spectrometry and then further confirmed with the Vitek 2 compact system. The clinical characteristics of patients, including age, sex, underlying diseases, infection type, use of invasive devices, and outcomes, were obtained from electronic medical records. We also calculated the Charlson comorbidity index (CCI) and Sequential Organ Failure Assessment (SOFA) score. The protocol for this study was approved by the Peking University Third Hospital Ethics Committee (M2021238), and the Guidelines for Human Experimentation People’s Republic of China (PRC) were followed throughout. Community-acquired infection (CAI), health care-associated infection (HCAI), and nosocomial infection (HAI) were identified as previously described (3).

### Antimicrobial susceptibility testing (AST).

All the ST65 *K. pneumoniae* strains isolated from the hospital were subjected to AST using the Vitek 2 system. The antimicrobial agents included ticarcillin/clavulanate (TCC), piperacillin/tazobactam (TZP), imipenem (IMP), meropenem (MEM), amikacin (AMK), tobramycin (TOB), aztreonam (ATM), cefepime (FEP), ceftazidime (CAZ), ciprofloxacin (CIP), levofloxacin (LEV), cefoperazone/sulbactam (CSL), trimethoprim/sulfamethoxazole (SXT), doxycycline (DOX), minocycline (MNO), and tigecycline (TGC). Ceftazidime/avibactam (CZA) and cefiderocol (FDC) were evaluated by the Kirby-Bauer (K-B method. The results were interpreted according to Clinical and Laboratory Standards Institute (CLSI) guidelines, while the breakpoint of TGC followed that of the FDA (11). Multidrug resistance was defined as resistance to three or more different antimicrobial classes, and extensive drug resistance was defined as susceptibility to only one or two classes, as previously described (3).

### Hypermucoviscosity.

The hypermucoviscous phenotype was defined by the string test (>5 mm), as described previously (20).

### Growth curve.

In brief, the strains were cultured overnight and then diluted to an optical density of ~0.1 at 600 nm (OD_600_). Then, the target strains were incubated at 37°C, and the OD_600_ was determined hourly for 16 h.

### Serum killing assay.

A serum killing assay was conducted to assess virulence *in vitro* according to previous studies. The concentration of bacteria was adjusted to 1 × 10^6^ CFU/mL. The 25-μL solution was mixed with 75 μL of serum from healthy volunteers and shaken at 37°C. Then, 1 μL of the mixture was spotted onto a blood agar plate at 0 h, 1 h, 2 h, and 3 h, and viable counts were recorded. The results were interpreted following a previous study (27). Each strain was tested three times.

### Biofilm formation assay.

The 0.5 McFarland bacterial suspension was prepared with 0.85% NaCl. A target bacterial solution of 10 μL was added to the 96-well plate, and then 190 μL of LB broth was added to each well. The 96-well plate was cultured in a 37°C incubator for 24 h, and then the supernatant was absorbed and discarded, and the plate was washed with aseptic distilled water 3 times. Then, we dyed the well plate with 200 μL of 0.1% crystal violet dye for 20 min and washed it with distilled water 3 times. Finally, we added 200 μL of 95% ethanol to each well, and the absorbance value of each well was read at 590 nm. The average value of the wells with the addition of only 200 μL of broth was set to acetyl (Ac). Each strain was tested three times, and the results were interpreted as previously described (28).

### Plasmid transferability and stability.

To explore the transferability of the plasmid harboring *bla*_KPC-3_, strain P1 was used as the donor, and sodium azide-resistant Escherichia coli J53 was used as the recipient. The donor and recipient isolates were cultured overnight at 37°C, and the monoclones were transferred to 5 mL of LB broth at 37°C for 16 h. Subsequently, the bacterial solutions were diluted with broth at a ratio of 1:100 and incubated at 37°C for 3 to 4 h. The donor and recipient strains were mixed in a 1:1 ratio and then cocultured at 37°C overnight. Finally, the mixture was spotted onto MacConkey agar containing meropenem (2 μg/mL) and sodium azide (200 μg/mL). After overnight culture, the transconjugants were finally screened. To further evaluate the stability of the plasmid harboring *bla*_KPC-3_, we continuously passaged to the 10th generation and then assessed AST.

### Galleria mellonella infection model.

Galleria mellonella larvae weighing 300 mg were randomly selected and grouped for killing assays, with 10 larvae per group. The *K. pneumoniae* strain concentration was adjusted to 1 × 10^6^ CFU/mL. The larvae were injected with 10 μL of bacterial suspension using a Hamilton syringe. The injected larvae were placed on aseptic plastic plates, and mortality rates were observed for 72 h. The experiments were repeated three times (21).

### Whole-genome sequencing and bioinformatics analysis.

All the DNA of the ST65 strains was prepared using the TIANamp bacterial DNA kit (Tiangen Biotech, Beijing, China), and then the whole genome was sequenced using the NovaSeq platform. In brief, low-quality reads were removed using FastQC. We trimmed the reads using fastp software (https://github.com/OpenGene/fastp). The remaining raw data were *de novo* assembled using SPAdes 3.13 and annotated using Prokka (29). Antimicrobial-resistant (AMR) genes, virulence genes, insertion sequences (IS), K and O locus serotypes, and plasmid replicon types were identified using ResFinder (30), the Virulence Factor Database (31), and the Isfinder (32), PlasmidFinder (33), and Kleborate (34) databases. To further demonstrate the genomic characteristics of MDR-ST65 hvKp (P1 and PEKP4222), nanopore sequencing was performed on the minION platform, and long reads were assembled with Illumina short reads using Unicycler (35).

To obtain the genetic context of *bla*_CTX-M-3_ within the ST65 strains, published data of the ST65 strains (up to August 2021) and their associated complete genomes from the NCBI database were analyzed in the study. We pairwise compared the ST65 genomes with K. pneumoniae HS11286 (accession number NC_016845) using MUMmer 3.2.3 to identify the single nucleotide polymorphisms (SNPs) and then combined the SNP sites according to the reference genome (K. pneumoniae HS11286) using a previously described method (https://github.com/generality/iSNV-calling). The recombination sites were further identified using Gubbins software (36). The concatenated sequences of filtered polymorphic sites conserved in all genomes (core genome SNPs, cgSNPs) were selected to perform phylogenetic analysis using the maximum likelihood method with FastTree software (37).

### Ethical approval statement.

All methods have been performed in accordance with the Declaration of Helsinki. The protocol for this study was approved by the Peking University Third Hospital Medical Science Research Ethics Committee (M2021545). Due to the retrospective nature of the study, the need for approval was waived by the Peking University Third Hospital Medical Science Research Ethics Committee, and all the patient data enrolled in this study were anonymized.

### Data availability.

The genome sequences in this study were deposited in the NCBI database under BioProject accession numbers PRJNA856039 and PRJNA838135. The data sets used and/or analyzed during the current study are available from the corresponding author on reasonable request.
