# Supplementary material for: Emergence of Extensively Drug-Resistant and Hypervirulent KL2-ST65 Klebsiella pneumoniae Harboring blaKPC-3 in Beijing, China
Source: Microbiol Spectr. 2022 Nov 14;10(6):e03044-22. doi: 10.1128/spectrum.03044-22 (PMC9769991; doi:10.1128/spectrum.03044-22)
Supplement: Supplemental file 1 — Fig. S1 to S3 and Tables S1 to S5. Download spectrum.03044-22-s0001.pdf, PDF file, 0.4 MB [file spectrum.03044-22-s0001.pdf]

Supplementary Figure 1. Growth curves of the ST65 strains.

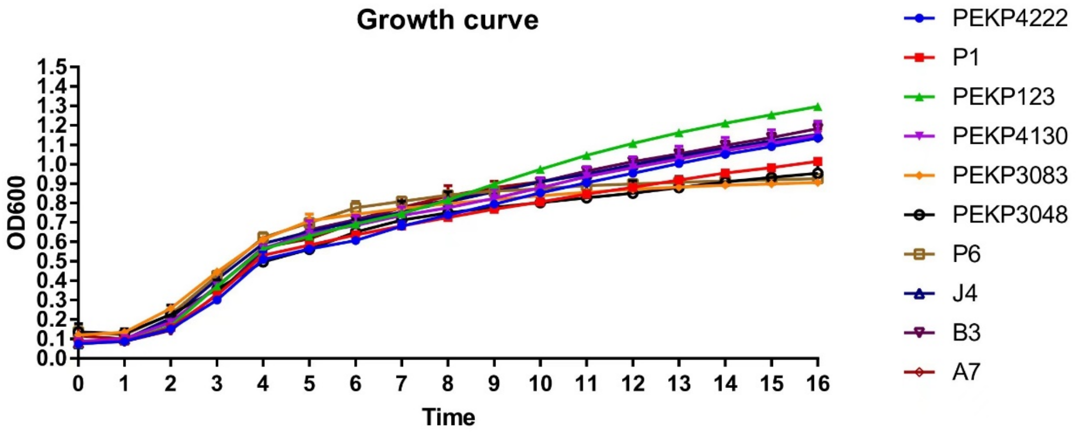

Supplementary Figure 2. Serum killing assays of the ST65 isolates.

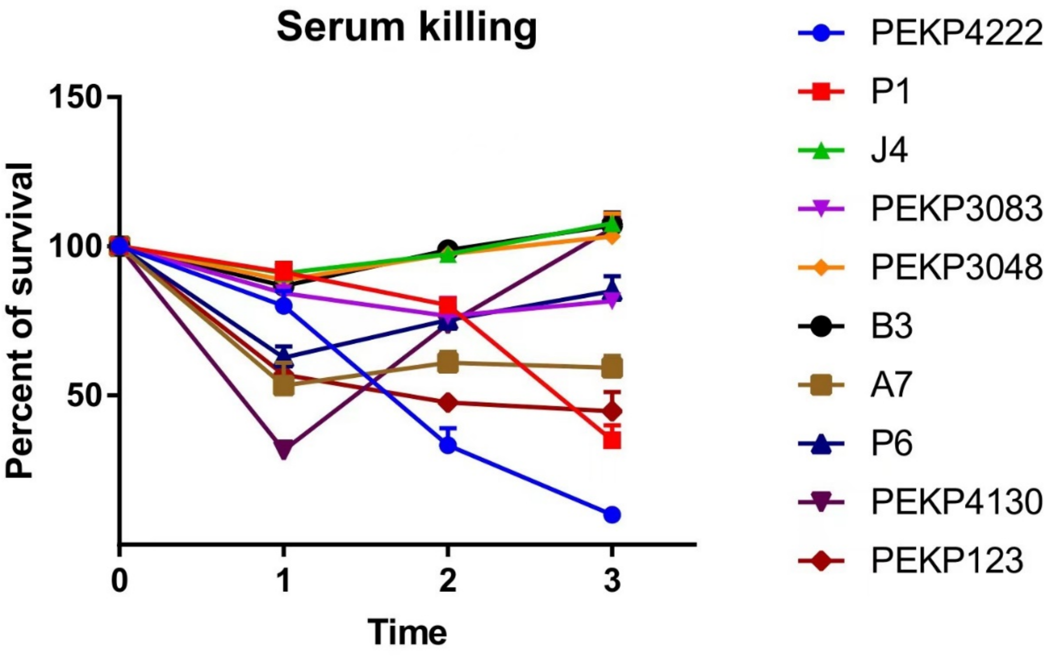

Supplementary Figure 3. The genomic differences amongst the ST65 isolates and the potential intra-host transmission route.

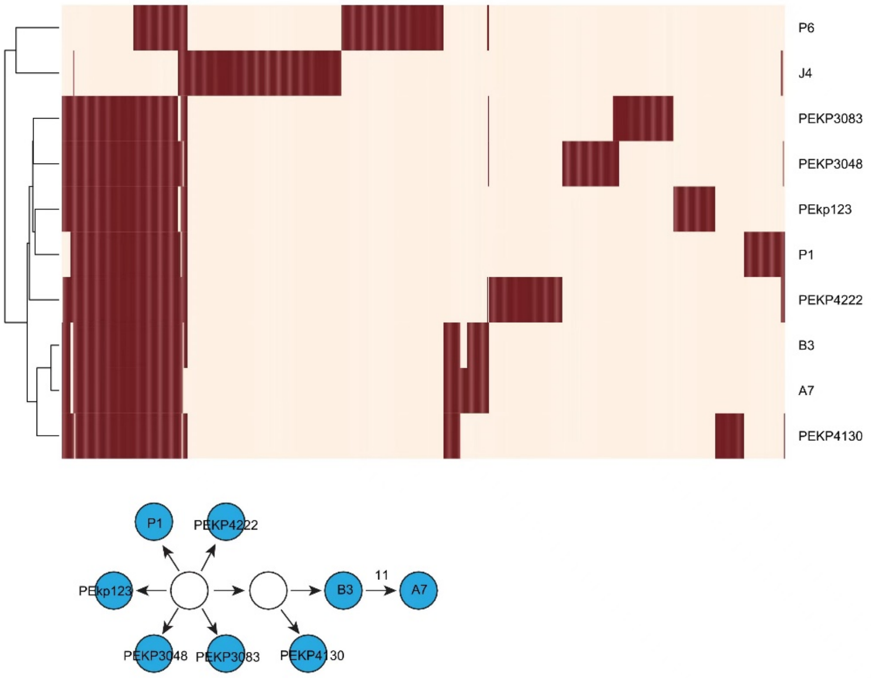

Supplementary Table 1. The crosstab of Serum Killing assay and virulence score.

	Serum Killing- Sensitive	Serum Killing- Resistant	Total
virulence_score=3	1	2	3
virulence_score=5	3	4	7
Total	4	6	10

P=1.000

Supplementary Table 2. The crosstab of String test and virulence score.

	String test- Positive	String test- Negtive	Total
virulence_score=3	3	0	3
virulence_score=5	6	1	7
Total	9	1	10

P=1.000

Supplementary Table 3. The crosstab of Biofilm assay and virulence score.

	Biofilm+	Biofilm++	Total
virulence_score=3	3	0	3
virulence_score=5	6	1	7
Total	9	1	10

P=1.000

Supplementary Table 4. The crosstab of Serum Killing assay and string test.

	Serum Killing- Sensitive	Serum Killing- Resistant	Total
String test-Positive	5	4	9
String test-Negtive	1	0	1
Total	6	4	10

P=1.000

Supplementary Table 5. The crosstab of Serum Killing assay and Biofilm assay.

	Serum Killing- Sensitive	Serum Killing- Resistant	Total
Biofilm+	5	4	9
Biofilm++	1	0	1
Total	6	4	10

P=1.000
